# Don’t Monkey with a Buzz-saw

**Published:** 1896-09

**Authors:** 


### DON'T MONKEY WITH A BUZZ-SAW.

Already we hear the rumblings of a threatened storm that has for its purpose a letting down of the bars of progress in veterinary legislation in the Empire State. Indistinctly we hear uncertain mutterings as to the severity of the laws governing veterinary science and practice in that great Commonwealth. Utterances as to the injustice of the law to a few have been heard, forgetful as it were of the fact that no law of any worth but what must for a time work hardships for some. But these latter are but for a period, and time and effort will correct; while the law of to-day is the foundation-stone of posterity for the veterinary profession, and every true devotee longs for its higher attainment, and when he has finished his work, wishes to see it better and stronger in every way than he found it. Don't temporize with a good law for which so hard a battle was waged! Don't try to fill it with loopholes! Don't endeavor to correct certain apparent defects that time will quickly efface! Don't endanger the whole superstructure by meddling with the foundation! Don't monkey with a buzz-saw that may prove worse than a two-edged sword and cut and destroy the whole fabric around which are to be woven every just and equitable safeguard for you and your successors in the pursuit of a calling that has honor for its chief gem, happiness and joy as shining attributes as a reward for the labor it imposes, and which brings sufficient of the world's goods to every true devotee to well fill all his daily needs, but when prostituted to the greed of gain alone loses every charm it possesses and leaves bitterness, hatred, and envy as the dregs in the cup of sorrow one drinks when he bends the profession of comparative medicine to such an unholy alliance. Forewarned is forearmed. Every man to his post. No quarter to those who would jeopardize or tear down that which has been built at so great a cost.

